# Surgery

**Published:** 1913-06

**Authors:** James Swain


### Progress of the Medical Sciences.

### SURGERY.

The existence of **cervical ribs** has received more attention from anatomists than clinicians, but the possibility of such an abnormality should always be considered in obscure cases of vascular, muscular or nervous disturbance in the upper limbs. In a recent discussion on this subject at the Royal Society of Medicine, Dr. Farquhar Buzzard[1] said that for some years he had been in the habit of submitting to an X-ray examination almost all cases in which pain in the arms was a predominant symptom, and as a result of his observations he found that not only did a cervical rib cause pain, distributed longitudinally on either the radial or ulnar sides of the limb, but also might produce a typical acroparæsthesia with pain limited to the hand and fingers.

Speaking from the anatomical point of view, Dr. Wood Jones[2] thinks it certain that the vascular symptoms in the arm are not produced in all cases by compression of the artery, but

[1] *Proc. Roy. Soc. Med.*, 1912–13, v., Clin. Sect., p. 139.

[2] *Ibid.*, p. 95.

that just as pressure on the lowest cord of the brachial plexus produces the muscular and sensory changes, so it may in some cases also produce the vascular symptoms. It is the compression of the vasomotor fibres in the lower cord of the plexus which causes these symptoms. Mr. Percy Sargent[1] says in some instances the injury to the nerves is brought about by the bony part of a long rib, but in other cases the nerves are in close relationship with the non-bony part of the anomalous rib, which consists of a dense fibrous cord embedded in muscle, attached above to the rudimentary bone whilst below it is most commonly attached to the first dorsal rib. As the sternum rises in inspiration the posterior part of the first dorsal rib tends to be depressed, so that the band, the upper attachment of which is immovable, is tightened. In this manner the nerves are damaged, and the possibilities of such damage are increased by excessive use of the arm. A study of the skiagrams of these cases shows that the degree of the symptoms depends in no way upon the size of the bony portion, and probably the direction of the bony point of the rib is more important than its size.

The symptoms of cervical rib may be divided into motor, sensory and vasomotor systems respectively. Dr. Kinneir Wilson[2] classes the sensory symptoms as subjective and objective. In the former group we have tingling, numbness and "pins and needles," referred either to the ulnar or radial side of the hand more frequently than to all the fingers, and mostly unilateral. It is rare to find the pain starting from the region of the neck or shoulder, but generally in the forearm, hand or fingers, and most patients say that it always radiates in a downward direction. Objective sensory symptoms are variable. In some cases no definite alteration in cutaneous sensibility can be discovered; in others there is a diminution or loss to all forms of cutaneous sensibility, the distribution of which is never exactly that of either the radial or ulnar nerves, and does not harmonise entirely with a root supply.

The motor phenomena are practically pathognomonic, and consist of two main types. The first of these, which may be designated the median type, is very frequent, and in it there is an early local wasting of the muscles of the thenar eminence, in which the muscles involved are the abductor pollicis and opponens pollicis alone, all the other thenar muscles, including the flexor brevis pollicis, being intact. The other type of muscular atrophy corresponds, roughly speaking, to an ulnar distribution; in other words, we find general wasting of the interossei and an approximation to the *main en griffe*. In most of the cases of this kind it will be found that the patient complains of paræsthesia along the ulnar border of the hand and in the ulnar fingers, whereas in the cases offering the local thenar

[1] *Ibid.*, p. 117. [2] *Ibid.*, p. 133.

atrophy of the first type the paræsthesiæ are on the radial side. Occasionally muscular cramps in the hand and fingers are noted by the patient without any muscular atrophy being discoverable.

When vasomotor phenomena are present the limb may be smaller than on the opposite side, the hand may be pale or blue and cold, the radial pulse may be absent and gangrene sometimes supervenes.

In an analysis of twenty-nine cases, Mr. Percy Sargent[1] found twenty-six females and three males, with an average age of about thirty-eight years. The first symptom noticed by the patient was as follows :—Pain, seventeen ; numbness, five ; swelling or coldness of the hand, four ; and clumsiness in the fingers, three. In nine instances the onset of symptoms was ascribed to the following causes :—Excessive sewing, two ; use of a sewing machine, one ; piano-playing, one ; house painting, two ; wearing a heavy overcoat, one ; lifting a patient, one ; and childbirth, one. In the last two the onset of symptoms was sudden. The symptoms for which the patient sought relief were :—Pain alone, four ; pain and wasting, nine ; pain and numbness, six ; wasting and numbness, five ; and wasting alone, five. In every case the abnormal ribs were bilateral, though rarely symmetrical ; the symptoms, however, were bilateral in only five of the twenty-nine patients. As a rule, when the symptoms were unilateral, the arm on the side of the smaller rib was affected ; thus in nineteen cases in which the relation was specially noted the symptoms were on the side of the larger rib in only eight.

Relief of the symptoms is obtained by removal of the abnormality, but the operation is not always easy. Sir Rickman J. Godlee[2] considers that most of the difficulties are avoided by making a transverse incision above the rib and not over it ; whereas Mr. Thorburn[3] considers that the operation is most easily performed through a long incision straight down and well back on the neck, over the anterior border of the trapezius muscle. The latter had only met with cases in which there were combined vascular and nervous symptoms, and had not been called upon to operate for vascular symptoms alone, though he recognised that an operation might be necessary where there was marked congestion or anæmia or threatened gangrene of the part. As a result of twenty cases, Mr. Thorburn found that all were greatly improved. Pain was cured in four-fifths of the cases which could be traced afterwards ; paralysis was cured in half the cases, though some weakness remained in the others ; anæsthesia was cured in all, though recovery from this was slow, and the sense of coldness never entirely disappeared.

Operation is, unfortunately, often immediately followed by symptoms of more or less severe, though usually transient,

[1] *Loc. cit.* [2] *Ibid.*, p. 140. [3] *Ibid.*, p. 113.

character. Dr. Howell[1] was able to follow up twenty-five cases in which thirty operations—as in five cases bilateral ribs were removed—were performed by several surgeons. Of the twenty-five patients operated on, of whom two only were males, there were symptoms due to operation in eighteen instances. Such symptoms were pain, more or less severe, in neck, shoulder or arm, and muscular weakness, which varied considerably both in extent and duration. In eighteen cases pain was complained of, in some instances being very severe. The duration of this symptom was, as a rule, from one to three months ; but in one case, in which there was a decidedly neurotic element, pain was still complained of seven years after operation. Fifteen patients showed motor symptoms as a result of operation. The usual complaint was of weakness of the whole arm, lasting from two weeks to three months, but in four cases the symptoms were more pronounced. In only twelve instances was there absolutely no motor or sensory disturbance following operation.

In dealing with the ultimate effects of operation, Dr. Howell divides the cases into four classes, according to the symptoms they exhibited :—1, vasomotor symptoms only, two cases ; 2, subjective sensory symptoms only, five cases ; 3, motor symptoms and subjective, but no objective sensory symptoms, eleven cases ; 4, complete cases, which include all the above symptoms, and exhibit in addition objective sensory disturbances. He summarises the results of operative treatment of these cases as follows :—In a large proportion of cases some symptoms, such as pain and weakness in the arm, may be expected to follow the operation, but not to last more than about three months. The vasomotor symptoms, which are present in nearly all cases, will be certainly improved, and in the majority of cases pain will be relieved or cured. With regard to weakness and atrophy, the expectation is that the operation, if not delayed too long, will greatly improve this condition. There is not, as a rule, complete restoration of the wasted muscles nor complete recovery from the vasomotor disturbance.

James Swain.

